# Antibiotic treatment adequacy and death among patients with Pseudomonas aeruginosa airway infection

**DOI:** 10.1371/journal.pone.0226935

**Published:** 2019-12-31

**Authors:** Josefin Eklöf, Kirstine Møller Gliese, Truls Sylvan Ingebrigtsen, Uffe Bodtger, Jens-Ulrik Stæhr Jensen

**Affiliations:** 1 Department of Internal Medicine, Section of Respiratory Medicine, Herlev and Gentofte Hospital, University of Copenhagen, Hellerup, Denmark; 2 Department of Nephrology, Rigshospitalet, University of Copenhagen, Copenhagen, Denmark; 3 Department of Respiratory Medicine, Hvidovre Hospital, University of Copenhagen, Copenhagen, Denmark; 4 Department of Respiratory Medicine, Naestved Hospital, University of Southern Denmark, Naestved, Denmark; 5 Department of Medicine, Zealand University Hospital Roskilde, Roskilde, Denmark; 6 Institute of Regional Health Research, University of Southern Denmark, Odense, Denmark; 7 Department of Infectious Diseases, CHIP & PERSIMUNE, Rigshospitalet, University of Copenhagen, Copenhagen, Denmark; Laurentian, CANADA

## Abstract

**Objective:**

The effect of antibiotics on survival in patients with pulmonary *Pseudomonas aeruginosa* is controversial. The aim of this study is to *i)* determine the prevalence of adequate antibiotic treatment of *P*. *aeruginosa* in an unselected group of adult non-cystic fibrosis patients and *ii)* to assess the overall mortality in study patients treated with adequate vs. non-adequate antibiotics.

**Methods:**

Prospective, observational study of all adult patients with culture verified *P*. *aeruginosa* from 1 January 2010–31 December 2012 in Region Zealand, Denmark. Patients with cystic fibrosis were excluded. Adequate therapy was defined as any antibiotic treatment including at least one antipseudomonal beta-lactam for a duration of at least 10 days. Furthermore, *P*. *aeruginosa* had to be tested susceptible to the given antipseudomonal drug and treatment had to be approved by senior clinician to fulfil the adequate-criteria.

**Results:**

A total of 250 patients were identified with pulmonary *P*. *aeruginosa*. The vast majority (80%) were treated with non-adequate antibiotic therapy. All-cause mortality rate after 12 months was 49% in adequate treatment group vs. 52% in non-adequate treatment group. Cox regression analysis adjusted for age, gender, bacteraemia, comorbidities and bronchiectasis showed no significant difference in mortality between treatment groups (adequate vs. non-adequate: hazard ratio 0.95, 95% CI 0.59–1.52, P = 0.82).

**Conclusion:**

Adequate antipseudomonal therapy was only provided in a minority of patients with pulmonary *P*. *aeruginosa*. Adequate therapy did not independently predict a favourable outcome. New research initiatives are needed to improve the prognosis of this vulnerable group of patients.

## Introduction

*Pseudomonas aeruginosa* is a non-fermentative Gram-negative bacillus frequently found in the airways in hospitalised patients with underlying chronic pulmonary disease [1,2].

Infection with *P*. *aeruginosa* plays a particular important role in cystic fibrosis (CF), where most patients acquire a chronic infection, which is associated with high morbidity and mortality [3].

While early antibiotic treatment directed against *P*. *aeruginosa* in CF has considerably improved the clinical condition and delaying onset chronic infection, the role of antibiotics against *P*. *aeruginosa* in other chronic pulmonary disease, as bronchiectasis or chronic obstructive pulmonary disease (COPD), is less clear [4–6]. Present guidelines of acute and chronic pulmonary infections with *P*. *aeruginosa* rest on low grade evidence due to lack of randomised interventional trials. Instead, most treatment recommendations are based on expert consensus and extrapolated from experience in CF [7,8].

European [9] and North American [10,11] guidelines for management of community and hospital acquired pneumonia advocate therapeutic selection based upon severity of infection and comorbid conditions, with an antipseudomonal beta-lactam drug as mono therapeutic cornerstone agent. In Denmark, a common and pragmatic treatment approach to *P*. *aeruginosa* infection is often intravenous antipseudomonal beta-lactam in combination with an adjunct antibiotic for 10–14 days.

The aim of this study is to estimate the number of adult non-CF patients treated for at least 10 days beta-lactam therapy against pulmonary *P*. *aeruginosa*, and to test the hypotheses that this regimen reduces mortality compared to patients treated with shorter treatment duration or other use of an antibiotic agent not active against *P*. *aeruginosa*.

## Materials and methods

Observational cohort study of all consecutive adult hospitalised patients with culture verified *P*. *aeruginosa* in lower respiratory tract specimens from 1 January 2010–31 December 2012 at the Department of Clinical Microbiology, Region Zealand, Denmark. Follow-up ended 31 December 2016. Patients below 18 years of age and patients with CF were excluded. Medical records were reviewed for patient data: demographics, comorbidities, current medication, bacteraemia and blood test markers (CRP and urea). Analyses were performed on data regarding the initial *P*. *aeruginosa*-positive sample in the study period.

For this study, the authors were granted access to microbiological data and clinical data in medical records in accordance with current Danish laws (Data Protection Agency: REG-14-2013). According to these, no informed patient consent or approval by The National Committee on Health Research Ethics is required.

Patients were categorised into two groups, depending on received antibiotic treatment:

1) Adequate treatment: defined as any antibiotic therapy including at least one antipseudomonal beta-lactam (e.g. piperacillin/tazobactam, ceftazidime or meropenem) for a minimum of 10 days and within 30 days after positive *P*. *aeruginosa*-sample. The following standard doses were considered adequate based on the national recommendation at the time for the present study: Piperacillin/tazobactam 4/0.5 every 8 hours, Ceftazidime 1 gram every 8 hours, Meropenem 2 gram every 8 hours. Furthermore, *P*. *aeruginosa* had to be tested susceptible to the given antipseudomonal drug and treatment had to be approved by senior clinician to fulfil the adequate-criteria. No further requirements or evaluation of the specific drug dosages were made by the study group.

2) Non-adequate treatment: defined as all other antibiotic treatment regimens, including no treatment.

### Outcomes

The primary outcome measure was all-cause mortality within 12 months. Secondary outcomes measure consisted of i) recurrence of *P*. *aeruginosa* during follow-up (microbiological failure) ii) length of first hospital stay (days). Post-hoc analyses were performed in a subgroup of patients treated for ≥ 14 days with dual antipseudomonal antibiotics, including at least one beta-lactam (e.g. piperacillin/tazobactam, ceftazidime or meropenem), and compared to all other treatment regimens.

### Statistical analyses

We used a Cox regression analysis of time to death and estimated hazards ratios and corresponding confidence intervals. Age, gender, bacteraemia, comorbidity (Charlson’s index of comorbidity) and bronchiectasis were included as covariates. Continuous data were analysed using median with corresponding interquartile range (ICR) and compared using two-sided non-parametric test (Mann-Whitney U test). Categorical data were analysed using frequency and percentage and compared between groups using two-sided Fischer’s exact test. P-values < 0.05 were considered significant. All analyses were performed using SAS v. 9.4 (SAS Institute Inc., Cary, NC, USA) and SPSS v. 22 (IBM SPSS Statistics).

## Results

A total of 253 patients were detected with culture verified *P*. *aeruginosa*. Two patients were excluded due to age below18 years and 1 patient was excluded due to known diagnosis of CF. The remaining 250 patients were diagnosed either by sputum sampling (66%), tracheal suction (26%) or bronchoalveolar lavage (8%). The majority of patients treated with adequate or non-adequate antibiotics were hospitalised (86% vs. 89%) and treated under the clinical picture of pneumonia (68% vs. 64%). Patient characteristics according to treatment group are summarised in Table 1.

**Table 1:** Patient characteristics of the study cohort.

	All patients	Patients treated with adequate antibiotic therapy	Patients treated with non-adequate antibiotic therapy
	(N = 250)	(N = 45)	(N = 205)
Age, years, median (IQR)	71 (63–79)	72 (63–77)	71 (65–79)
Male, gender, n (%)	135 (54%)	24 (53%)	91 (44%)
Airway disease, n (%)			
Asthma	14 (6%)	2 (4%)	12 (6%)
Bronchiectasis	28 (11%)	8 (18%)	20 (10%)
COPD	136 (54%)	24 (53%)	112 (55%)
Cancer, all cause, n (%)	16 (6%)	2 (4%)	14 (7%)
CCI, median (IQR)	2 (1–3)	2 (1–3)	2 (1–3)
Inhaled corticosteroid, n (%)	153 (61%)	30 (67%)	123 (60%)
Bacteraemia, n (%)	18 (7%)	3 (7%)	15 (7%)
Blood marker, median (IQR)			
CRP (mg/L)	122 (47–231)	167 (57–275)	115 (47–219)
Urea (mmol/L)	6.8 (3.3–12.3)	7.1 (5.1–11.1)	6.7 (2.7–12.6)

Abbreviations: IQR, interquartile range; COPD, chronic obstructive pulmonary disease; CCI, charlson’s index of comorbidity, CPR; C-reactive protein.

### Treatment

Adequate treatment with antipseudomonal beta-lactam was provided in 45/250 (18%) patients with median treatment duration of 13 days (IQR 12–14). In 40/45 (89%) patients the beta-lactam treatment was supplemented with an adjunct antibiotic of either ciprofloxacin (36/40 (90%) patients) or aminoglycoside (4/40 (10%) patients). The median duration of ciprofloxacin was 13 days (interquartile range (IQR) 8–15). Adequate treatment was initiated within a median of 1 day after a positive *P*. *aeruginosa* sample (IQR 0–5).

In the non-adequate treatment group, 21/205 (10%) patients received beta-lactams as monotherapy for a median of 3 days (IQR 2–5). In total, 33/205 (16%) patients were treated with a combination of beta-lactam and ciprofloxacin for a median duration of 5 (IQR 3–7) respectively 7 days (IQR 4–14). Nineteen percent (38 patients) received ciprofloxacin as monotherapy with a median treatment period of 9 days (IQR 5–10). The remaining 113/205 (55%) patients did either not receive antibiotics or were treated with antibiotics lacking *P*. *aeruginosa*-activity.

### Outcomes

**Fig 1:**
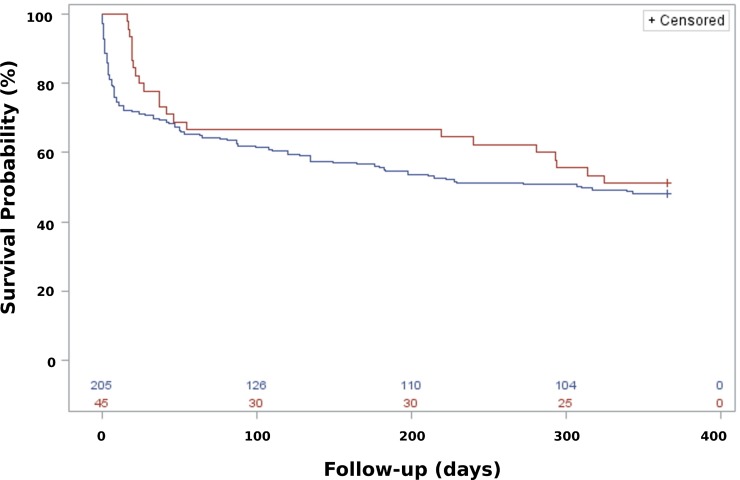
Unadjusted Kaplan-Meier survival estimates by treatment group in patients with pulmonary *Pseudomonas aeruginosa*. Red line: adequate antibiotic treatment. Blue line: non-adequate antibiotic treatment. Log-rank test: p = 0.41.

The primary outcome, all-cause mortality after 12 months, was high and similar in both the adequate and non-adequate group (49% vs. 52%). Fig 1 illustrates Kaplan-Meier survival plot.

No significant difference was observed between the groups in the unadjusted or multivariable adjusted Cox regression analyses (Table 2).

**Table 2:** Association between antibiotic treatment and all-cause mortality in cox regression model.

	Unadjusted model		Multivariate adjusted model	
All-cause mortality after 12 months	Hazard ratio (95% CI)	P-value	Hazard ratio (95% CI)	P-value
Adequate antibiotic treatment vs. non-adequate antibiotic treatment	0.83 (0.52–1.31)	0.42	0.95 (0.59–1.52)	0.82

Multivariate model was adjusted for age, bacteraemia, bronchiectasis, charlson’s index of comorbidity index and gender.

**Fig 2:**
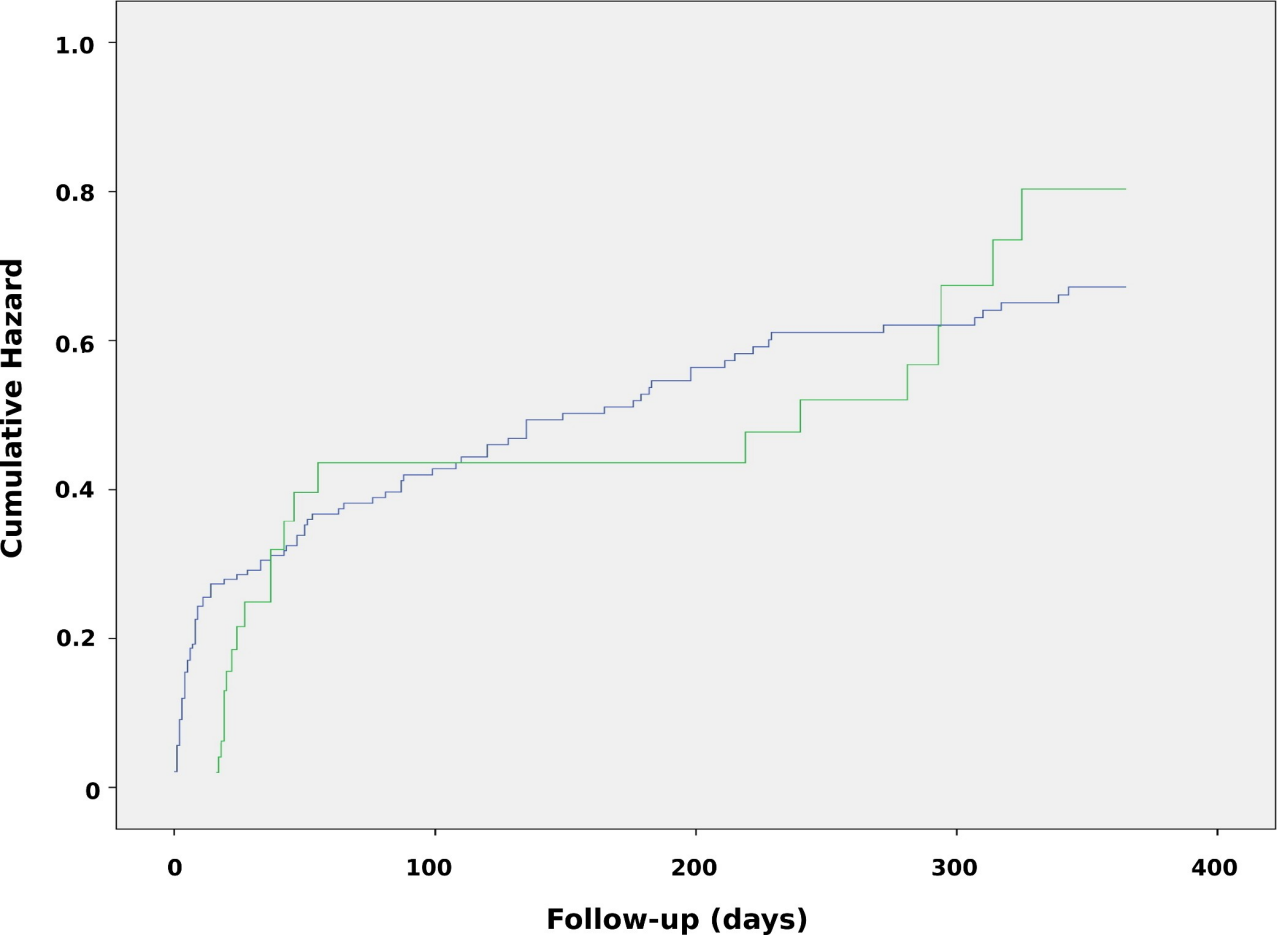
Adjusted cumulative hazard plot by treatment group in patients with pulmonary *Pseudomonas aeruginosa*. Green line: adequate antibiotic treatment. Blue line: non-adequate antibiotic treatment.

Fig 2 shows the cumulative hazard for outcome.

Increasing age, bacteraemia and higher comorbidity score were independently associated with mortality. The mortality rates were compared to a large reference population of 1.897 *P*. *aeruginosa-negative* patients hospitalised for exacerbation of COPD and there was markedly lower overall mortality in the reference group (30% vs. 51%). Patient characteristics are shown in S1 Table.

The length of hospital stay was significantly longer for patients treated adequately compared non-adequately treated (18 vs. 5 median days, IQR 3–24 vs. 2–11; p < 0.0001). Moreover, the incidence of recurrent *P*. *aeruginosa*-positive sputum culture within the study period was not lower in the adequate treatment group (42% vs. 32%; p = 0.22).

### Post-hoc analyses

**Fig 3:**
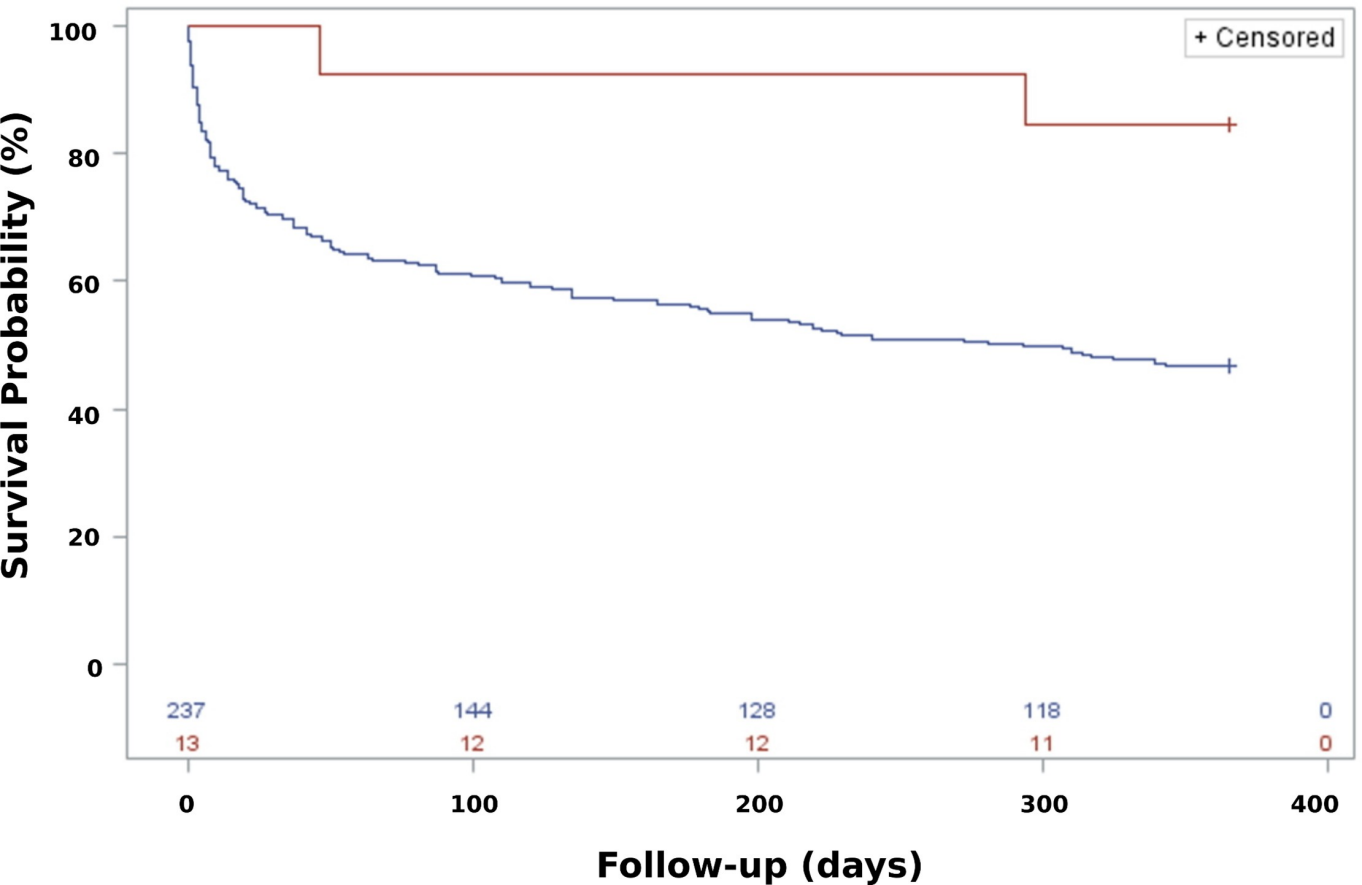
Unadjusted Kaplan-Meier survival estimates by treatment group in patients with pulmonary *Pseudomonas aeruginosa*. Red line: ≥ 14-days dual antibiotic treatment. Blue line: all other antibiotic treatment regimes. Log-rank test: p = 0.016.

A total of 13/250 (5%) patients were treated with dual antipseudomonal antibiotics for a minimum of 14 days. The mortality rate after 12 months was 15% (2/13 patients) vs. 53% (126/237 patients); Fig 3 illustrates Kaplan-Meier survival plot.

No association was seen between dual therapy and reduced mortality when adjusting for covariates in the Cox regression analysis (Table 3). Age, bacteraemia and comorbidity remained as significant predictors of outcome.

**Table 3:** Association between antibiotic treatment and all-cause mortality in post-hoc cox regression model.

	Unadjusted model		Multivariate adjusted model	
All-cause mortality after 12 months	Hazard ratio (95% CI)	P-value	Hazard ratio (95% CI)	P-value
≥ 14-days dual antibiotic treatment vs. all other antibiotic treatment regimes.	0.21 (0.053–0.86)	0.03	0.34 (0.083–1.39)	0.13

Multivariate model was adjusted for age, bacteraemia, bronchiectasis, charlson’s index of comorbidity index and gender.

## Discussion

In a sample of 250 adult non-CF patients with *P*. *aeruginosa* lung infection, adequate antipseudomonal therapy was only provided for a minority of the patients and was not associated with lower mortality compared to non-adequate therapy.

Our study reports markedly higher mortality rate, independent of treatment group, compared to previous literature in the respiratory field [12–15]. An explanation could be inclusion of patients with bronchiectasis and more severe stage of COPD, which is closely related to presence of pulmonary *P*. *aeruginosa* [16,17] and increased risk of mortality [18,19]. However, the overall mortality rate was higher when compared to a large cohort of patients hospitalised with COPD exacerbation (S1 Table). Information regarding severity of pulmonary comorbidities, including lung function test and chest computed tomography (CT), would be helpful to explore this possible scenario in the study population. Influence by multidrug resistant *P*. *aeruginosa*, which also has been related to poor prognosis [15], cannot be ruled out. However, the overall incidence of such *P*. *aeruginosa* is low in Denmark, why any substantial influence must be considered highly unlikely (S2 Table).

Few well-controlled studies have evaluated the optimal antibiotic treatment of *P*. *aeruginosa* in adult non-CF patients, both inside and outside of the hospital, and the potential clinical advantages of different treatment regimens has been a controversial subject for many years [20–24]. To our knowledge, there are no present data making direct comparisons between shorter or longer treatment durations and clinical outcome in patients with bronchiectasis or COPD. General recommendations for acute infectious exacerbations (any bacterial pathogen) are usually 5–14 days [7,25].

In Denmark, *P*. *aeruginosa* infection in bronchiectasis and COPD patients are typically treated with a 10–14 days’ course of antibiotics including a pseudomonas-active beta-lactam. Based on this, a lower limit of 10 days was set as minimum criteria for the duration of beta-lactam in the present study. The median duration of 13 days of beta-lactam thus corresponds well with the current standard of duration.

The potential clinical benefit of combination therapy over monotherapy for *P*. *aeruginosa* has also been a subject of discussion over the last decades. Commonly cited reasons in favour of combination therapy is the possibility of minimising the development of resistant bacterial strains and increasing the likelihood of therapeutic success through antimicrobial synergy. Although the Kaplan-Meier plot (Fig 2) was somewhat indicative of a difference, a multivariable confounder adjusted analysis did not support this. However, we cannot rule out that the way we define our exposure of adequate versus non-adequate treatment regimen could potentially bias interpretations.

Despite our interesting findings, the study also has important limitations which need to be taken into consideration when interpreting the results. First, the study is an observational study. Due to the non-randomised design, the results cannot determine causality between treatment and outcome. A potential limitation is confounding by indication since treatment is highly likely to be influenced by the severity of disease. Thus, it is possible, that the adequate regimen is given to the patients with most severe clinical state, and less favourable prognosis, while less sick patients, with better prognosis, could be expected to be treated less aggressively. The adequately treated patients might therefore have worse or, at best, similar prognosis as the non-adequately treated patients. On the contrary, a severe state might also lead to early discontinuation of treatment, and thereby non-adequate duration. However, the significant longer hospital admission among the adequately treated patients suggest that the first argument is more likely to be the case in our study. Moreover, we have not registered the administered antibiotic dosage on patient level. Although we expect the adequate antibiotic treatment regimens to be given according to national guidelines, we cannot be completely certain of this as the study group made no independent assessment of the drug administrations. This is a major limitation. However, *P*. *aeruginosa* had to be tested susceptible to the given antipseudomonal drug and treatment had to be approved by senior clinician to fulfil the adequate-criteria. Data regarding resistance was not registered but is expected to be low based on microbiological data from the Capital Region in Denmark (S2 Table).

Secondly, the study population is inhomogeneous. This challenges group comparisons and makes it difficult to draw a definite conclusion based on the results. However, the sample size in the current study is larger than in most comparable studies. Another strength is the consecutive selection of patients, which reduces the risk of selection bias.

## Conclusion

Adequate antipseudomonal therapy was only provided in a minority of patients with pulmonary *P*. *aeruginosa*. Overall mortality rates were high in both treatment groups and adequate therapy did not independently predict a favourable outcome. Thus, our data support the clinical impression that lung infections with *P*. *aeruginosa* are complex. New research initiatives are needed to improve the prognosis of this vulnerable group.

## Supporting information

S1 Table Patient characteristics of the study population and reference population: The reference population consists of 1.897 *P*. *aeruginosa*-negative COPD outpatients hospitalised with exacerbation of COPD in Region Zealand and the Capital Region in Denmark in 2010–2012. (DOCX)

S2 Table Antibiotic resistance in *P*. *aeruginosa* during 2005–2013 in the Capital Region of Denmark. Data are based on test results from the Department of Clinical Microbiology at Herlev and Hvidovre University Hospital.
(DOCX)

## References

[1] Arancibia F, Bauer TT, Ewig S, Mensa J, Gonzalez J, Niederman MS, et al Community-acquired pneumonia due to gram-negative bacteria and Pseudomonas aeruginosa: Incidence, risk, and prognosis. Arch Intern Med. 2002;162(16):1849–58. 10.1001/archinte.162.16.1849 12196083

[2] Holm JP, Hilberg O, Noerskov-Lauritsen N, Bendstrup E. Pseudomonas aeruginosa in patients without cystic fibrosis is strongly associated with chronic obstructive lung disease. Dan Med J. 2013;60(6):1–5. 23743112

[3] Folkesson A, Jelsbak L, Yang L, Johansen HK, Ciofu O, Hoiby N, et al Adaptation of Pseudomonas aeruginosa to the cystic fibrosis airway: An evolutionary perspective. Nat Rev Microbiol. 2012;10(12):841–51. 10.1038/nrmicro2907 23147702

[4] Martínez‐Solano L, Macia MD, Fajardo A, Oliver A, Martinez JL. Chronic Pseudomonas aeruginosa Infection in Chronic Obstructive Pulmonary Disease. Clin Infect Dis. 2008;47(12):1526–33. 10.1086/593186 18990062

[5] Murphy TF, Brauer AL, Eschberger K, Lobbins P, Grove L, Cai X, et al Pseudomonas aeruginosa in chronic obstructive pulmonary disease. Am J Respir Crit Care Med. 2008;177(8):853–60. 10.1164/rccm.200709-1413OC 18202344

[6] Rakhimova E, Wiehlmann L, Brauer AL, Sethi S, Murphy TF, Tümmler B. Pseudomonas aeruginosa Population Biology in Chronic Obstructive Pulmonary Disease. J Infect Dis. 2009;200(12):1928–35. 10.1086/648404 19919307

[7] Polverino E, Goeminne PC, McDonnell MJ, Aliberti S, Marshall SE, Loebinger MR, et al European Respiratory Society guidelines for the management of adult bronchiectasis. Eur Respir J. 2017;50(3). 10.1183/13993003.00629-2017 28889110

[8] Pasteur MC, Bilton D, Hill AT. British Thoracic Society guideline for non-CFbronchiectasis. Thorax. 2010;65(Suppl 1):i1–58. 20627931 10.1136/thx.2010.136119

[9] Woodhead M, Blasi F, Ewig S, Garau J, Huchon G, Ieven M, et al Guidelines for the management of adult lower respiratory tract infections—Full version. Clin Microbiol Infect. 2011;17:E1–59. 10.1111/j.1469-0691.2011.03672.x PMC7128977 21951385

[10] Mandell LA, Wunderink RG, Anzueto A, Bartlett JG, Campbell GD, Dean NC, et al Infectious Diseases Society of America/American Thoracic Society Consensus Guidelines on the Management of Community-Acquired Pneumonia in Adults. Clin Infect Dis. 2007;44(Supplement 2):S27–72. 17278083 10.1086/511159 PMC7107997

[11] Kalil AC, Metersky ML, Klompas M, Muscedere J, Sweeney DA, Palmer LB, et al Management of Adults With Hospital-acquired and Ventilator-associated Pneumonia: 2016 Clinical Practice Guidelines by the Infectious Diseases Society of America and the American Thoracic Society. Clin Infect Dis. 2016;63(5):e61–111. 10.1093/cid/ciw353 27418577 PMC4981759

[12] Renom F, Yáñez A, Garau M, Rubí M, Centeno MJ, Gorriz MT, et al Prognosis of COPD patients requiring frequent hospitalization: Role of airway infection. Respir Med. 2010;104(6):840–8. 10.1016/j.rmed.2009.12.010 20106648

[13] Finch S, McDonnell MJ, Abo-Leyah H, Aliberti S, Chalmers JD. A comprehensive analysis of the impact of Pseudomonas aeruginosa colonization on prognosis in adult bronchiectasis. Ann Am Thorac Soc. 2015;12(11):1602–11. 10.1513/AnnalsATS.201506-333OC 26356317

[14] Loebinger MR, Wells AU, Hansell DM, Chinyanganya N, Devaraj A, Meister M, et al Mortality in bronchiectasis: A long-term study assessing the factors influencing survival. Eur Respir J. 2009;34(4):843–9. 10.1183/09031936.00003709 19357155

[15] Montero M, Domínguez M, Orozco-Levi M, Salvadó M, Knobel H. Mortality of COPD patients infected with multi-resistant pseudomonas aeruginosa: A case and control study. Infection. 2009;37(1):16–9. 10.1007/s15010-008-8125-9 19139809

[16] Patel IS, Seemungal TA, Wilks M, Lloyd-Owen SJ, Donaldson GC, Wedzicha JA. Relationship between bacterial colonisation and the frequency, character, and severity of COPD exacerbations. Thorax. 2002;57(9):759–64. 10.1136/thorax.57.9.759 12200518 PMC1746426

[17] Miravitlles M. Relationship Between Bacterial Flora in Sputum and Functional Impairment in Patients With Acute Exacerbations of COPD. CHEST J. 1999;116(1):40. 10.1378/chest.116.1.40 10424501

[18] Groenewegen KH, Schols AMWJ, Wouters EFM. Mortality and mortality-related factors after hospitalization for acute exacerbation of COPD. Chest. 2003;124(2):459–67. 10.1378/chest.124.2.459 12907529

[19] Connors AF, Dawson NV, Thomas C, Harrell FE, Desbiens N, Fulkerson WJ, et al Outcomes following acute exacerbation of severe chronic obstructive lung disease. The SUPPORT investigators (Study to Understand Prognoses and Preferences for Outcomes and Risks of Treatments). Am J Respir Crit Care Med.1996;154:959–67. 10.1164/ajrccm.154.4.8887592 8887592

[20] Garnacho-Montero J, Sa-Borges M, Sole-Violan J, Barcenilla F, Escoresca-Ortega A, Ochoa M, et al Optimal management therapy for Pseudomonas aeruginosa ventilator-associated pneumonia: An observational, multicenter study comparing monotherapy with combination antibiotic therapy. Crit Care Med. 2007;35(8):1888–95. 10.1097/01.CCM.0000275389.31974.22 17581492

[21] Park SY, Park HJ, Moon SM, Park KH, Chong YP, Kim MN, et al Impact of adequate empirical combination therapy on mortality from bacteremic Pseudomonas aeruginosa pneumonia. BMC Infect Dis. 2012;12(1):1. 23157735 10.1186/1471-2334-12-308 PMC3519646

[22] Llor C, Moragas A, Hernández S, Bayona C, Miravitlles M. Efficacy of Antibiotic Therapy for Acute Exacerbations of Mild to Moderate Chronic Obstructive Pulmonary Disease. Am J Respir Crit Care Med. 2012;186(8):716–23. 10.1164/rccm.201206-0996OC 22923662

[23] Anthonisen NR, Manfreda J, Warren CP, Hershfield ES, Harding GK, Nelson NA. Antibiotic therapy in exacerbations of chronic obstructive pulmonary disease. Ann Intern Med. 1987;106(2):196–204. 10.7326/0003-4819-106-2-196 3492164

[24] Chastre J, Wolff M, Fagon J-Y, Chevret S, Thomas F, Wermert D, et al Comparison of 8 vs 15 days of antibiotic therapy for ventilator-associated pneumonia in adults: a randomized trial. JAMA. 2003;290(19):2588–98. 10.1001/jama.290.19.2588 14625336

[25] GOLD- Global initirativ for chronic obstrucitve lung disease. 2018 New GOLD reports. http://goldcopd.org/. Date last assessed: April 1 2018.

