# Supplementary material for: Antibiotic treatment adequacy and death among patients with Pseudomonas aeruginosa airway infection
Source: PLoS One. 2019 Dec 31;14(12):e0226935. doi: 10.1371/journal.pone.0226935 (PMC6938358; doi:10.1371/journal.pone.0226935)
Supplement: S1 Table — (DOCX) [file pone.0226935.s001.docx]

| **S1 Table. Patient characteristics of the study population and reference population:** | | |
| --- | --- | --- |
| **The reference population consists of 1.897 *P. aeruginosa*-negative COPD outpatients hospitalised with exacerbation of COPD in Region Zealand and the Capital Region in Denmark in 2010-2012.** | | |
|  | **Study population, all patients** | **Reference population, all patients** |
|  | **(N=250)** | **(N=1.897)** |
|  |  |  |
| All-cause mortality after 12 months, n (%) | 128 (51%) | 569 (30%) |
| Age, years, median (IQR) | 71 (63-79) | 70.1 (63.4-76.9) |
| Male, gender, n (%) | 135 (54%) | 867 (46%) |
| Airway disease, n (%) |  |  |
| Asthma | 14 (6%) | 185 (10%) |
| Bronchiectasis | 28 (11%) | 14 (0.1%) |
| COPD | 136 (54%) | 1.897 (100%) |
| Cancer, all cause, n (%) | 16 (6%) | 220 (12%) |
| CCI, median (IQR) | 2 (1-3) | N/A |
| Inhaled corticosteroid, n (%) | 153 (61%) | 1.573 (83%) |
| Bacteraemia, n (%) | 18 (7%) | N/A |
| Blood marker, median (IQR) |  |  |
| CRP (mg/L) | 122 (47-231) | N/A |
| Urea (mmol/L) | 6.8 (3.3-12.3) | N/A |
|  |  |  |
| *Abbreviations: IQR, interquartile range; COPD, chronic obstructive pulmonary disease; CCI, charlson’s index of comorbidity,* | | |
| *CPR; C-reactive protein, N/A; not available.* |  |  |
