# Supplementary material for: Antibiotic treatment adequacy and death among patients with Pseudomonas aeruginosa airway infection
Source: PLoS One. 2019 Dec 31;14(12):e0226935. doi: 10.1371/journal.pone.0226935 (PMC6938358; doi:10.1371/journal.pone.0226935)
Supplement: S2 Table — Data are based on test results from the Department of Clinical Microbiology at Herlev and Hvidovre University Hospital. (DOCX) [file pone.0226935.s002.docx]

| **S2 Table. Antibiotic resistance in *P. aeruginosa* during 2005-2013 in the Capital Region of Denmark. Data are based on test results from the Department of Clinical Microbiology at Herlev and Hvidovre University Hospital.** | | |
| --- | --- | --- |
| **Antibiotic:** | **Performed test, n:** | **Frequency of antibiotic resistance, n (%):** |
| Piperacillin/Tazobactam | 10.920 | 485 (4.4) |
| Ceftazidime | 10.640 | 519 (4.9) |
| Meropenem | 10.850 | 384 (3.5) |
| Ciprofloxacin | 10.994 | 946 (8.6) |
| Gentamycin | 10.283 | 170 (1.7) |
